# Patients' Payments

**Published:** 1894-04-14

**Authors:** 


### The Institutional Workshop.

### HOSPITAL FINANCE.

### V.—PATIENTS' PAYMENTS.

Deducting the four sources of hospital income already discussed, viz., the State, the commune or rates, endowments, and voluntary offerings, there still remains one sufficient in itself to maintain many hospitals in good working order without any form of assistance. This final source, hitherto almost entirely neglected in England, is the payments of patients. It will be well to examine the practice of other countries in this respect before considering the causes which have led English hospitals, with few exceptions, to refuse any form of payment from their inmates.

"In America the majority of patients treated in the hospitals occupy pay beds or paying wards; there is relatively little free medical relief anywhere, and the number of hospital beds in proportion to the population is insignificant as compared with those to be found in England and the older countries of Europe." * Taking seventeen of the larger hospitals of the United States, and examining their income for the years 1889 and 1890, it was found that patients' payments produced 26·56 and 21·17 per cent. of the respective totals. In respect to out-patients, free relief appears to be given there also to a quite unnecessary and harmful extent, owing mainly to the demand for clinical material.

In Switzerland the hospitals, with one or two exceptions, are on the pay system, but the patients' fees, which vary from 8d. to 12s. per diem, do not of course defray all expenses. The deficit is made up mainly by private contributions and State subsidies.

In Norway and Sweden those who can afford it are expected in all the hospitals to pay a proportion of the expense incurred on their behalf. The system of graduated payments is admirably worked out, and the abuse of free medical relief is reduced to a minimum.

In Italy the scheme of maintenance varies largely in different parts of the country. Patients' fees form, however, in almost every case a considerable proportion of the total receipts, and this is the more noteworthy in view of the grinding poverty of the Italian peasants and the richness of the charitable endowments of the country.

Comparing one country with another, it becomes clear that the amount contributed by the poor towards their own support under hospital treatment does not in any degree depend upon their rate of wages or their capability to spare the money. On the contrary, where wages are low and even a sufficiency of food is not always procurable, the thriftiest habits are found often to exist and some provision for ill-health to be forthcoming. The following figures show what percentage of the total income of the hospitals of the United Kingdom is formed by patients' payments:—

| | London. | Provincial. | Scotch. | Irish. |
|---|---|---|---|---|
| General Hospitals ... | 1·14 ... | 1·17 ... | 3·13 ... | 7·14 |
| Special Hospitals ... | 6·70 ... | 10·76 ... | 0·18 ... | 6·58 |

It will be seen that in London, where the rate of wages is highest, the proportion is lowest. In Ireland, where wages are low and poverty extreme, the patients pay something like seven times as much as in London towards their general treatment. All who have had opportunties of investigating the circumstances of the persons who may be said to form the great out-patients' class of the metropolis, are well aware that in a large proportion of cases payments would be readily forthcoming were it required. The objections to payment rest, in fact, on a far deeper basis than the poverty of the patients, although the public tone has been so completely enervated in later years by free medical treatment that considerable opposition would no doubt be stirred up by any attempt to enforce payment. The difficulties in the way of receiving payment for in-patients fall under three heads. There is, first, the difficulty of the subscriber, who gives his money for a charity, and might, it is thought, withhold his contribution if the benefit conferred were not of the nature of an entirely free gift. In answer to this, it may be urged that right principles of charity are every day becoming more firmly established among us; the benefit conferred which calls out a corresponding effort on the part of the person benefited is seen to be of a higher order than that which checks all attempts at self-dependence, and demands only grateful submission. If some few subscribers were lost in the endeavour to place the hospitals on a more wholesome basis of thrift, many more who now stand aloof in grave disapproval of the present system would supply their place. Free wards there must always be, too, in every hospital worthy of the name, for it cannot of course be pretended that payment should under all circumstances be required. The second difficulty which deters hospital managers from accepting payment is more serious. They are hampered by the necessity for providing clinical material for the medical schools, and the fear is that free access for purposes of instruction could not be had to patients from whom even a small payment had been received. The opening of the poor law infirmaries for clinical instruction would do much to obviate this difficulty, and would largely extend the available field of observation. The free wards would remain at the service of the students, and it is believed that in practice, such is the odd vanity of disease, few objections would be made by the paying patients to clinical teaching where carried out in a suitable and considerate manner.

Lastly, it is felt in many quarters that the reception of payment is an open acknowledgment that the hospital is not being reserved exclusively for those destitute persons for whom it was primarily intended. If they can pay, it is argued, they have no business here, where they crowd out others more in need. And, moreover, if they can pay, it inflicts a grave injustice upon the doctors that they should receive gratuitous attendance in a hospital instead of paying their own medical attendant in their own homes. This is indeed a difficult and delicate problem. It must not, however, be supposed that the reception of payment from patients would call it into being. The problem is already one of the most pressing which hospital authorities, in the rapidly altering conditions of our social life, are called upon to solve, and its full consideration lies beyond the scope of this article. That the hospitals and free dispensaries already compete with the smaller medical practitioners to an overwhelming extent is undeniable. But it is not by any means clear that the enforcement of payment from those who can afford it would tend to increase the evil. Rather it might be expected to place a serious check on the abuse of our medical charities by that not insignificant class of individuals who never dream of paying for what they can get for nothing. One consequence must inevitably follow the acceptance of patients' fees, and that is the payment of those medical men attached exclusively to the hospital service. But this is a reform urgently called for on other accounts, and one which cannot long be delayed.

The importance to hospital finance of the contributions of patients may be estimated by the calculation that an average sum of 2s. a day on one-third of the hospital beds of London would produce just about the additional £100,000 which it is estimated should be found yearly in order to place the metropolitan hospitals on a sound financial basis. That the average London workman should be able to pay this sum for either himself or any member of his family when disabled by sickness may be thought by some an impossible supposition. In the country, however, far higher sums are habitually found by the labouring classes for medical attendance, even when the wages average no more than from 12s. to 15s. a week. Sickness is, unhappily, almost as inevitable an adjunct of family life as rent, but owing to the demoralising effect of our London charities, it is habitually regarded by the bulk of the working classes as a remote contingency, in face of which something may be confidently expected to "turn up." Something *does* turn up. Free dispensaries and out-patients' departments abound for the curing of minor ailments and the gift of gigantic bottles of "doctor's stuff." In grave illness all the resources of modern science are placed at the sick man's disposal, and a skilled physician, whose income very probably falls short of that of his patient, provides him with free attendance. Meantime his family is in receipt of an allowance from church or chapel or other organisation (possibly more than one), where the plea that the bread-winner is disabled, and that nothing is coming in, is undoubtedly one of the most respectable which can be urged. With these inducements to improvidence, is it to be wondered at that the London workman, with three or four times the amount of wages of his fellow-labourer in the country, is found in the hour of sickness absolutely unprepared to meet any of its charges? And is it not one of the gravest questions of our time whether a more bracing system should not be attempted which may relieve at once the financial strain on the hospitals, and replace the bulk of the people in a state of healthy dependence on their own efforts?

